# How FGF23 shapes multiple organs in chronic kidney disease

**DOI:** 10.1186/s40348-021-00123-x

**Published:** 2021-09-18

**Authors:** Maren Leifheit-Nestler, Dieter Haffner

**Affiliations:** Department of Pediatric Kidney, Liver and Metabolic Diseases, Pediatric Research Center, Hannover Medical School Children’s Hospital, Carl-Neuberg-Str. 1, 30625 Hannover, Germany

**Keywords:** Chronic kidney disease, Fibroblast growth factor 23, Bone, Cardiovascular diseases, Left ventricular hypertrophy, Cognition

## Abstract

Chronic kidney disease (CKD) is associated with distinct alterations in mineral metabolism in children and adults resulting in multiple organ dysfunctions. Children with advanced CKD often suffer from impaired bone mineralization, bone deformities and fractures, growth failure, muscle weakness, and vascular and soft tissue calcification, a complex which was recently termed CKD-mineral and bone disorder (CKD-MBD). The latter is a major contributor to the enhanced cardiovascular disease comorbidity and mortality in these patients. Elevated circulating levels of the endocrine-acting phosphaturic hormone fibroblast growth factor (FGF) 23 are the first detectable alteration of mineral metabolism and thus CKD-MBD. FGF23 is expressed and secreted from osteocytes and osteoblasts and rises, most likely due to increased phosphate load, progressively as kidney function declines in order to maintain phosphate homeostasis. Although not measured in clinical routine yet, CKD-mediated increased circulating levels of FGF23 in children are associated with pathological cardiac remodeling, vascular alterations, and increased cognitive risk. Clinical and experimental studies addressing other FGF23-mediated complications of kidney failure, such as hypertension and impaired bone mineralization, show partly conflicting results, and the causal relationships are not always entirely clear. This short review summarizes regulators of FGF23 synthesis altered in CKD and the main CKD-mediated organ dysfunctions related to high FGF23 levels.

## Fibroblast growth factor 23 in chronic kidney disease

**Fig. 1:**
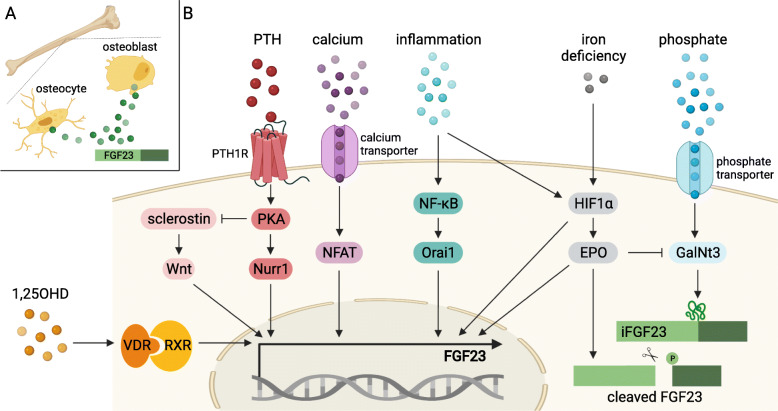
Regulators of FGF23 in CKD. **a** Fibroblast growth factor (FGF) 23 is expressed and secreted in the bone by osteocytes and osteoblasts. **b** In CKD, enhanced parathyroid hormone (PTH) levels stimulate the transcription of *FGF23* by binding to PTH 1 receptor (PTH1R) activating protein kinase A (PKA)/nuclear receptor related-1 (Nurr1) signaling pathway. In addition, PTH-mediated PKA activation inhibits sclerostin that leads to induction of the Wnt pathway following *FGF23* mRNA expression. Treatment with 1,25-dihydroxy vitamin D (1,25OHD) in CKD patients upregulates *FGF23* transcription via binding to vitamin D receptor (VDR)/retinoid X receptor (RXR) heterodimer complex that translocates into the nucleus and activates vitamin D response elements (VDRE) in the *FGF23* gene. Furthermore, *FGF23* transcription is stimulated by calcium-dependent nuclear factor of activated T cells (NFAT) signaling pathway leading to induction of NFAT response elements in the FGF23 promotor. High phosphate induces polypeptide n-acetylgalactosaminyltransferase 3 (GalNt3), thereby stabilizing intact FGF23 (iFGF23) protein on the post-translational level. Iron deficiency and inflammation stabilize hypoxia-inducible factor 1 α (HIF1α) that bind to HIF-binding sites in the FGF23 promotor stimulating its transcriptional activity. Moreover, HIF1α mediates the cleavage of FGF23 protein through erythropoietin (EPO), while EPO itself enhances *FGF23* transcription. The increase in CKD-related inflammatory factors stimulates nuclear factor kappa-light-chain-enhancer of activated B cells (NF-kB) mediating *FGF23* transcription through Orai1 activation. Created with BioRender.com

Fibroblast growth factor (FGF) 23 is a bone-derived phosphaturic hormone (Fig. 1a) that acts on the kidney via FGF receptor (FGFR) 1/Klotho-mediated downregulation and internalization of sodium-phosphate cotransporters NaPi2a and NaPi2c decreasing phosphate reabsorption and finally serum phosphate levels. FGF23 suppresses renal synthesis of 1,25-dihydroxy vitamin D (1,25OHD) that leads to reduced dietary phosphate absorption in the intestine further lowering serum phosphate concentration. In addition, FGF23 inhibits parathyroid hormone (PTH) expression and secretion in the parathyroid glands [1].

Chronic kidney disease (CKD) is a complex, debilitating condition that affects over 70 million people worldwide. In CKD, the above-mentioned parameters of mineral metabolism are dysregulated with FGF23 being the first altered biochemical parameter. Increased FGF23 levels are already noted in patients with CKD stage 2. Increasing with declining glomerular filtration rate (GFR), FGF23 is responsible for maintaining phosphate homeostasis. If the expression of Klotho as a co-factor of FGF23 in the kidney decreases during CKD progression, a renal FGF23 resistance develops. In combination with reduced viable nephron number, the renal excretory capacity of phosphate is no longer sufficient despite high FGF23 and consequently serum phosphate levels rise. Increasing phosphate with concomitant reduced 1,25OHD levels leads to hypocalcemia that stimulates PTH secretion resulting in secondary hyperparathyroidism (sHPT) [2]. PTH stimulates bone resorption and thereby enhances serum phosphate and calcium levels. The latter mechanism is thought to contribute to vascular calcification. FGF23, already increased in early stages of CKD, has a pivotal role in the complex changes in mineral metabolism during the progressive decline in kidney function leading to metabolic bone disease and enhanced cardiovascular (CV) morbidity summarized under the term CKD-mineral and bone disorder (CKD-MBD). In addition to the important beneficial effects of FGF23 in CKD stimulating phosphate excretion, even in children, high FGF23 levels are also associated with the progression of CKD and adverse secondary diseases, such as pathological cardiac remodeling, vascular alterations, and increased cognitive risk.

## Regulators of FGF23 in CKD

FGF23 is regulated on a transcriptional level and by post-translational modifications. The latter determines whether FGF23 is present as a full-length biologically active protein or cleaved fragments. The ratio between intact and cleaved FGF23 is thought to define its bioactivity, although the biological function of cleaved FGF23 fragment is still under investigation.

Phosphate, the critical factor in CKD, is discussed as a major regulator of its phosphaturic hormone FGF23 in the circulation. However, experimental studies in bone cell cultures and rodents show no induction of *FGF23* mRNA transcription due to high phosphate while others do. A recent study in UMR106 osteoblast-like cells demonstrate on molecular level that high phosphate does not stimulate *FGF23* expression but the stabilization of intact FGF23 protein via upregulation of polypeptide n-acetylgalactosaminyltransferase 3 (GalNt3) (Fig. 1b) [3]. These data indicate that rising serum phosphate levels in CKD may not induce *FGF23* transcription but result in increased circulating intact FGF23 concentrations contributing to enhanced renal phosphate excretion.

PTH, the second phosphaturic hormone increased in CKD, is shown to enhance *FGF23* transcription mediating high circulating FGF23 level in experimental renal failure [4]. Using UMR106 osteoblast-like cells, PTH binds to PTH 1 receptor (PTH1R) and activates protein kinase A (PKA) resulting in upregulation of nuclear receptor related-1 (Nurr1) mRNA expression and finally stimulation of *FGF23* transcription (Fig. 1b). Furthermore, the activation of PKA causes suppression of sclerostin that leads to induction of Wnt pathway. Finally, Wnt stimulates *FGF23* mRNA expression in bone (Fig. 1b). Since PTH rises in moderate to severe CKD, it may further enhance the already high circulating FGF23 level.

In CKD, high FGF23 suppresses 1,25OHD synthesis resulting in vitamin D deficiency; thus, vitamin D supplementation is a common treatment in those patients. UMR106 osteoblast-like and MC3T3-E1 osteocyte-like cells stimulated with 1,25OHD show an upregulation of *FGF23* transcription that is confirmed in animal experiments as well. 1,25OHD binds to vitamin D receptor (VDR) building a heterodimer with the retinoid X receptor (RXR) that translocates into the nucleus and binds to vitamin D response elements (VDRE) in corresponding target genes, such as FGF23 (Fig. 1b) [5]. Although vitamin D is shown to have organ protective properties, treatment with both active or native vitamin D is shown to further stimulate FGF23 synthesis in the bone that may further promote FGF23-associated complications in CKD patients.

Dietary phosphate restriction and treatment with phosphate binders are used to lower circulating phosphate and thereby FGF23 levels in CKD. However, only calcium-free phosphate binders are shown to reduce FGF23 in CKD patients while calcium-containing phosphate binders do not. Indeed, in MC3T3-E1 osteocyte-like cells, calcium stimulates *FGF23* transcription that is also confirmed in wild-type mice (Fig. 1b). The promotor of FGF23 has two binding sites for the transcription factor nuclear factor of activated T cells (NFAT); thus, NFAT is identified as a local regulator of *FGF23* expression that is upregulated in the bone of CKD mice due to calcium and inflammatory stimuli [6], suggesting an induction of *FGF23* mRNA expression. Interestingly, FGF23 itself activates the calcium-dependent calcineurin/NFAT pathway in cardiac myocytes promoting pathological cardiac remodeling [7], and cardiac myocyte-specific calcineurin transgenic mice show elevated FGF23 with severe left ventricular hypertrophy (LVH) [8], indicating a connection between activated calcineurin/NFAT signaling pathway and high FGF23 levels.

Besides the above-mentioned classical FGF23 regulators involved in altered CKD-related mineral metabolism, experimental research focuses on non-classical regulators, such as anemia [9]. Anemia is common in CKD and rises with CKD progression. The mechanism beyond is complex including decreased erythropoietin (EPO) production, iron deficiency, inflammation, and enhanced hepcidin levels, all of which are involved in the regulation of FGF23 transcription and/or post-translational modification (Fig. 1b). In a CKD mouse model, anemia increases FGF23 that can be rescued by treatment with EPO. Hepcidin-mediated inflammation-induced iron deficiency in mice stimulates *FGF23* transcription and causes increased levels of cleaved FGF23 fragments while intact FGF23 protein remains unchanged. In experimental CKD, iron deficiency enhances also intact FGF23 levels [10], suggesting that besides induction of *FGF23* mRNA, iron deficiency only causes high intact FGF23 concentrations when cleavage is impaired as in CKD. Several studies show that pro-inflammatory markers, such as interleukin 1β (IL-1β), tumor necrosis factor-alpha (TNF-α), and lipopolysaccharide (LPS), stimulate *FGF23* transcription in vivo and in vitro, although circulating intact FGF23 levels are not affected. Thereby, the nuclear factor kappa-light-chain-enhancer of activated B cells (NF-kB) mediates enhanced *FGF23* transcription through Orai1 activation (Fig. 1b) [9]. In addition, iron deficiency and inflammation stabilize hypoxia-inducible factor 1 α (HIF1α) that bind to HIF-binding sites in the promotor of FGF23 stimulating its transcriptional activity (Fig. 1b) [10]. Moreover, HIF1α mediates FGF23 cleavage through EPO (Fig. 1b). Many regulators of FGF23 transcription and processing have been discovered in the last years. As mentioned above, all these factors are dysregulated in CKD and thus may affect FGF23 transcription and the concentration of intact and cleaved FGF23. Early studies point out that only intact FGF23 is biologically active and can exert endocrine function. Although few, recent studies indicate that the proteolytically cleaved C-terminal fragment of FGF23 competitively inhibits the binding of full-length intact FGF23 to the FGFR1/Klotho complex and thus downregulates FGF23 signaling, indicating that the carboxy terminal tail of FGF23 also has a biological function [11]. Taken all these into account, the altered regulation of FGF23 transcription and cleavage affects its function. However, more research is needed to elucidate whether both classical and non-classical regulators of FGF23 biology are affected in CKD and may alter FGF23 transcription, post-translational modification and cleavage, and thereby its function.

## FGF23 and the progression of CKD

**Fig. 2:**
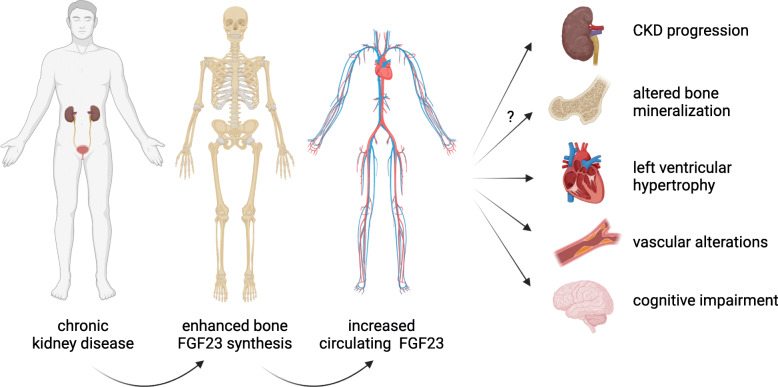
Secondary diseases caused by high FGF23 in CKD. Patients with chronic kidney disease (CKD) show enhanced synthesis of fibroblast growth factor (FGF) 23 in the bone leading to increased FGF23 in the circulation. High FGF23 in CKD promotes CKD progression, left ventricular hypertrophy, vascular alterations, and cognitive impairment. Whether or not FGF23 contributes to mineralization defects in CKD is still unclear. Created with BioRender.com

Starting in 2007, a study in 227 adult CKD patients found a significant association between circulating concentrations of biologically active intact FGF23 with estimated GFR (eGFR) across all stages of renal dysfunction at baseline [12]. After a median follow-up of 53 months, only baseline eGFR and FGF23 remain significant predictors for CKD progression in a multivariable analysis. Patients with FGF23 levels above a certain cut-off level obtained by ROC analysis show shorter progression times and a worse renal prognosis. In the following, similar results were found in different cohorts of patients, e.g., chronic IgA nephropathy and diabetic kidney disease. In a large community-based study, including over thirteen thousand individuals participating within the community-based Atherosclerosis Risk in Communities (ARIC) study, higher baseline FGF23 levels are associated with increased risk of developing end-stage kidney disease (ESKD) after a follow-up of up to 21 years even after adjustment for demographic characteristics, baseline eGFR, traditional risk factors for CKD, and markers of mineral metabolism [13]. In 419 children with CKD aged 1–16 years, one-third reach the progression endpoint requiring kidney replacement therapy after a median of 5.5 years [14]. Thus, FGF23 is an independent risk factor for CKD progression in children and adults with CKD (Fig. 2).

By performing a comparative transcriptome analysis in kidneys of mouse CKD models with elevated FGF23, Dai et al. identified FGF23-responsive transcripts associated with kidney damage and chronic inflammation, such as lipocalin-2, also known as neutrophil gelatinase-associated lipocalin (NGAL), transforming growth factor-beta (TGF-β), and TNF-α [15]. Chronic inflammation is common in CKD associated with poor outcome, and therefore, it is suggested that FGF23 further contributes to the progression of CKD via the induction of inflammatory markers. Vice versa, as mentioned above, inflammation stimulates FGF23 synthesis in osteocytes [10] and may thereby induce a vicious circle interconnecting FGF23 with inflammation promoting CKD progression.

## FGF23 and impaired bone mineralization in CKD

Bone mineralization defects are even prevalent in children with early CKD stages and highly prevalent in children on dialysis. Several FGF23-associated bone diseases are described, and although it is shown that FGF23 is a direct inhibitor of bone mineralization and its overexpression associates with osteomalacia in individuals with normal kidney function, it is unclear how FGF23 contributes to altered skeletal histology in CKD. In murine CKD-MBD, the CKD-driven increase in ostoblastic and osteocytic FGF23 synthesis contributes to mineralization defects and accumulation of the mineralizing inhibitor pyrophosphate in bone [16]. In a pediatric CKD cohort treated with peritoneal dialysis, higher FGF23 concentration is associated with decreased osteoid thickness and shorter osteoid maturation time but not with bone formation rate. In pre-dialysis CKD children, impaired skeletal mineralization is not related to enhanced circulating FGF23 levels. Cultured primary osteoblasts from CKD patients show impaired maturation and mineralization that is not affected by treatment with FGF23 in vitro [17]. In a cross-sectional study in adult CKD stage 5D patients, high FGF23 is negatively associated with mineralization lag time and osteoid maturation time. However, regression analysis shows that FGF23 is the only independent predictor of mineralization lag time. Overall, it must be further investigated whether or not FGF23 directly contributes to altered bone histomorphometric parameters in CKD (Fig. 2).

## FGF23 and left ventricular hypertrophy in CKD

Patients with CKD are six times more likely to die from CV causes than to develop ESKD. Cardiovascular disease (CVD) in pediatric ESKD is responsible for up to 36% of deaths [18]. LVH is present in 50–90% of adult patients with mild to severe CKD, respectively, and a leading cause of enhanced CVD within all CKD stages. The Cardiovascular Comorbidity in Children with CKD (4C) study monitors 15–48% LVH in children with increasing prevalence with CKD progression from CKD stages 3a to 5, respectively [19]. Prospective studies show an association between high FGF23 levels and greater risk of CV events (Fig. 2) and mortality in adult and pediatric CKD patients. In 2009, it was published that high FGF23 concentrations are independently associated with increased LV mass index and risk of LVH in pre-dialysis patients. Investigations in 3070 patients of the Chronic Renal Insufficiency Cohort (CRIC) study with a median eGFR of 42 ml/min/1.73 m^2^ demonstrate an independent association of high circulating FGF23 with LVH [7]. After initial clinical evidence of a link between high FGF23 levels and LVH in CKD nearly a decade ago, FGF23 is experimentally shown to phosphorylate phospholipase C gamma (PLCγ) via binding to FGFR isoform 4 on cardiac myocytes independent of Klotho. PLCγ phosphorylation leads to the activation of the calcineurin/NFAT signaling cascade and thereby induces prohypertrophic gene programs in vitro and in vivo [7, 20].

To date, a variety of other mechanisms have been experimentally addressed showing that enhanced FGF23 promotes ventricular arrhythmias, impaired Ca^2+^ signaling, activation of intra-cardiac renin–angiotensin–aldosterone system (RAAS), and LV fibrosis in vitro and in vivo. Translating these experimental findings into bedside, it is shown that intra-cardiac FGF23/FGFR4 expression is increased in pediatric ESKD patients developing LVH and is associated with a larger cross-sectional area of cardiac myocytes and higher expression of prohypertrophic B-type natriuretic peptide [21], but not with cardiac fibrosis. In 26 pediatric patients on dialysis, log-transformed circulating FGF23 concentrations are associated with increased LV mass index and higher interventricular septum thickness, while there is no association between FGF23 and LVH in pre-dialysis children [22]. Nevertheless, high FGF23 levels are associated with an increased prevalence of LVH in larger pediatric CKD cohorts enrolled in the Chronic Kidney Disease in Children (CKiD) study [14, 23]. Despite the induction of pathological cardiac remodeling due to increased FGF23 in CKD, the relationship between high FGF23 levels and the development of hypertension is unclear. Even in children with CKD prior to dialysis, the prevalence of hypertension is up to 47%. Experimental data suggest that FGF23 increases sodium reabsorption via upregulation of the amiloride-sensitive epithelial sodium channel (ENaC) in the renal collecting tubule and may thereby cause hypertension. The potential contribution of elevated FGF23 on the development of hypertension in the setting of CKD has been recently discussed in detail by Freundlich et al. [24].

## Vascular effects of high FGF23 in CKD

An impaired vascular system is a further characteristic of CKD. Mediators related to metabolic changes during uremia contribute to multiple functional and structural changes in blood vessels, including neointimal hyperplasia, coronary artery calcification, and calcification of arterial media resulting in ventricular fibrillation and increased pulse wave velocity, respectively. In the 4C study, carotid intima-media thickness increases in nearly 42% of children irrespective of CKD stage, suggesting vascular calcification [19].

Despite its multifactorial etiology, FGF23 is thought to contribute to excessively enhanced CV risk in patients with advanced CKD (Fig. 2). The first detectable vascular abnormality even in patients with mild CKD is endothelial dysfunction that is associated with oxidative stress, while such a link with FGF23 needs to be proven. In adult patients with CKD stages 3–4, elevated FGF23 levels are shown to be independently associated with decreased flow-mediated vasodilatation. In contrast, in patients on peritoneal dialysis, neither an association of high FGF23 with impaired endothelial function nor reduced arterial elasticity is observed. Unfortunately, experimental in vitro and ex vivo studies even show conflicting results regarding the impact of FGF23 on endothelial cell function.

In CKD-MBD, abnormal deposition of calcium-phosphate salts in blood vessels, valves, and heart causes vascular calcification, and clinical studies show that vascular calcification is an independent predictor for enhanced CV mortality in CKD. Local mineral depositions in vessels lead to intimal calcification reflecting atherosclerosis, medial calcification that induces vessel stiffness, increased pulse wave velocity, and finally LVH, and valvular calcification causing valve stenosis that can lead to heart failure and sudden cardiac death [25]. Active inducers of calcification in CKD are hyperphosphatemia, hypercalcemia, sHPT, inflammatory cytokines, and oxidative stress among others, whereby high phosphate is the strongest and most important stimulator of vascular calcification within all CKD stages. The role of FGF23 on vascular calcification is conflicting. Physiological FGF23 concentrations mediate their biological effects in a Klotho-dependent manner, while it is described that extremely high FGF23 exerts its pathological function in a Klotho-independent manner. Moreover, it was shown that re-expression of endogenous Klotho synthesis in the kidney or supplementation of exogenous soluble Klotho mitigates vascular calcification in CKD. The latter suggests that, if any, it is not FGF23 excess alone but rather the disturbed balance between FGF23 and its co-factor Klotho that contributes to the increased calcification in CKD. Clinical studies on this topic are rare and studies in cultures vascular smooth muscle cells and mouse aortic rings show either no effects of FGF23 on matrix calcification in the presence or absence of its co-receptor Klotho or phosphate, or an induction of phosphate-induced calcification by FGF23. Other reports even show a protective role of FGF23 on vascular calcification in vitro [25]. Additional insights come from studies in patients with hereditary hypophosphatemic rickets due to FGF23 excess. X-linked hypophosphatemia which is due to mutations in the phosphate regulating endopeptidase homolog X-linked (*PHEX*) gene is associated with elevated FGF23 serum levels, but there is no evidence for cardiovascular calcifications except patients receive excessive doses of oral phosphate promoting tertiary hyperparathyroidism [26, 27]. In contrast, autosomal-recessive hypophosphatemic rickets type 2 due to ectonucleotide pyrophosphatase/phosphodiesterase 1 (*ENPP1*) variants is associated with vascular calcifications. However, these are thought not to be due to FGF23 excess but rather as a consequence of the reduced synthesis of inorganic pyrophosphate (PPi), a well-known inhibitor of calcium hydroxyapatite crystal deposition and preventor of ectopic soft tissue calcification [28]. Taken together, it is still questioned whether high FGF23 levels alone are a major driver of vascular alterations in CKD, i.e., endothelial dysfunction and vascular calcification.

## FGF23 and cognitive impairment in CKD

Cognitive impairment is frequent in patients with CKD. The current knowledge of the pediatric brain in CKD was recently summarized by Harshman and Hooper, discussing, among others, hypertension to be a key clinical biomarker associated with poorer cognitive outcome in CKD children [29]. Although experimental data suggests that FGF23 may cause hypertension, a direct relationship between cognition and altered mineral metabolism is controversially discussed. FGF23 may pass through the blood–cerebrospinal fluid barrier, and together with FGFRs and its co-factor Klotho, FGF23 is expressed within the central nervous system (CNS), indicating that FGF23 may target the CNS and interfere cognition (Fig. 2). Indeed, overexpression of FGF23 in mice causes spatial memory deficits and a persistent strengthening of synapses in the hippocampus, reflecting an altered cellular correlate of learning and memory. Mice deficient for FGF23 show cognitive impairment, too, but without defects in gross structure or development of the brain and no changes in hippocampal synaptic plasticity [30]. In primary murine hippocampal cell culture, FGF23 enhances synaptic density independent of Klotho and stimulates primary neurite number with reduced arborization, suggesting less complex neuron morphology. Klotho is required for multiple hippocampal activities, delays age-related loss of neurogenesis, and preserves cognitive function, while Klotho deficiency causes premature neurogenic aging, synaptic changes, and impaired spatial memory [30]. Together, these experimental studies indicate a possible mechanism of altered memory formation in FGF23 excess and Klotho deficiency, such as in CKD.

A recent published study including 702 children enrolled in the CKiD study shows that high FGF23 concentrations are associated with lower performance in tests of executive function with regard to attention regulation [31]. In 263 adult hemodialysis patients, elevated FGF23 levels are associated with worse performance on a composite memory score, even after adjustment for measures of mineral metabolism. In contrast, a study in 605 patients with advanced CKD, investigating 25OHD, 1,25OHD, PTH, and FGF23, suggests that dysregulation in mineral metabolism does not contribute to impaired cognitive function in these patients. A single-center cohort study comparing 20 healthy individuals with 58 patients on chronic hemodialysis, sub-grouped in patients with normal cognitive function (NCF) and those with mild cognitive decline (MCD), shows significant differences between circulating FGF23 and Klotho levels in hemodialysis patients compared to healthy controls, but not between patients with NCF and MCD, suggesting that hemodialysis-related MCD is not related to altered FGF23 and Klotho [32]. Finally, a cross-sectional observational study in 69 hemodialysis patients recently demonstrated an association of cognitive impairment with intracranial artery calcification and low FGF23. Although the question whether FGF23 plays a direct role in cognitive impairment is not solved yet, cognitive dysfunctions occur in almost all stages of CKD and need to be carefully addressed in further studies.

## Conclusions and future perspectives

The management of pediatric CKD is challenging, and although it improves over the past years, comorbidities and mortality are still higher than in healthy children. Causes of disease are multifactorial, and thus, the identification of risk factors for CKD progression, CVD, and brain-associated disorders is of high interest. Modification and treatment of those risk factors early in CKD are important to reduce or even slower comorbidity progression to improve long-term quality of life for these children. Metabolic bone disease is thought to contribute to endothelial dysfunction, vascular calcification, and finally cardiac and brain dysfunction. Besides high phosphate levels, sHPT, vitamin D, and Klotho deficiency, increasing FGF23 plasma concentration is an independent predictor of the development of LVH in children with CKD. Further CVD risk factors in CKD are inflammation, anemia, volume overload, and hypertension, all of which are discussed to regulate FGF23 biology or be regulated by high FGF23. Cognitive deficits are observed in early childhood and develop in early CKD. Among others, high FGF23 is suggested to be a CKD-specific risk factor for vascular damage, neuronal toxicity, and blood–brain barrier disruption, all of that can cause stroke and small vessel disease and finally impact cognitive impairment.

However, no uniform guidelines for treatment strategies to reduce CKD-associated comorbidities are available. Independent of that whether or not targeting markers of abnormal mineral and bone metabolism in CKD, such as FGF23, will improve CVD and brain outcomes in children is not clear. There is a need for long-term pediatric clinical trial data evaluating cognitive and CVD outcomes over time with respect to CKD progression and clinical biomarkers. It is recommended that clinical research includes healthy control groups as well as a control group with other chronic disease entities to clearly attribute CVD and brain changes to the presence of CKD. The goal must be to better understand CKD-associated comorbidities, identify associated risk factors, and improve therapies to enhance the quality of life of CKD children and promote successful transition into adulthood.

## Data Availability

Not applicable.

## Abbreviations

1,25OHD: 1,25-Dihydroxy vitamin D
4C: Cardiovascular Comorbidity in Children with CKD
ARIC: Atherosclerosis Risk in Communities
CKD: Chronic kidney disease
CKD-MBD: Chronic kidney disease-mineral bone disorder
CKiD: Chronic kidney disease in children
CNS: Central nervous system
CRIC: Chronic renal insufficiency cohort
CV: Cardiovascular
CVD: Cardiovascular disease
eGFR: Estimated glomerular filtration rate
ENaC: Epithelial sodium channel
ENPP1: Ectonucleotide pyrophosphatase/phosphodiesterase 1
EPO: Erythropoietin
ESKD: End-stage kidney disease
FGF: Fibroblast growth factor
FGFR: Fibroblast growth factor receptor
GalNt3: n-Acetylgalactosaminyltransferase 3
GFR: Glomerular filtration rate
HIF1α: Hypoxia-inducible factor 1 alpha
IL-1β: Interleukin 1 beta
LPS: Lipopolysaccharide
LVH: Left ventricular hypertrophy
MCD: Mild cognitive decline
NCF: Normal cognitive function
NFAT: Nuclear factor of activated T cells
NF-κB: Nuclear factor kappa-light-chain-enhancer of activated B cells
NGAL: Neutrophil gelatinase-associated lipocalin
Nurr1: Nuclear receptor related-1
PHEX: Phosphate regulating endopeptidase homolog X-linked
PKA: Protein kinase A
PLCγ: Phospholipase C gamma
PPi: Inorganic pyrophosphate
PTH: Parathyroid hormone
PTH1R: Parathyroid hormone 1 receptor
RAAS: Renin–angiotensin–aldosterone system
RXR: Retinoid X receptor
sHPT: Secondary hyperparathyroidism
TGF-β: Transforming growth factor-beta
TNF-α: Tumor necrosis factor-alpha
VDR: Vitamin D receptor
VDRE: Vitamin D response element
